# Supplementary material for: Visualizing band selective enhancement of quasiparticle lifetime in a metallic ferromagnet
Source: Nat Commun. 2021 Dec 9;12:7169. doi: 10.1038/s41467-021-27277-6 (PMC8660887; doi:10.1038/s41467-021-27277-6)
Supplement: Supplementary file 1 — Supplementary Information [file 41467_2021_27277_MOESM1_ESM.pdf]

Supplementary Information for “Visualizing band selective enhancement of quasiparticle lifetime in a metallic ferromagnet”

Na Hyun Jo,[1,2,*] Yun Wu,[1,2,*] Thaís V. Trevisan,[1,2] Lin-Lin Wang,[1] Kyungchan Lee,[1,2] Brinda Kuthanazhi,[1,2] Benjamin Schrunk,[1,2] S. L. Bud'ko,[1,2] P. C. Canfield,[1,2] P. P. Orth,[1,2] and Adam Kaminski[1,2,†]

[1] *Division of Materials Science and Engineering, Ames Laboratory, Ames, Iowa 50011, USA*

[2] *Department of Physics and Astronomy, Iowa State University, Ames, Iowa 50011, USA*

(Dated: November 3, 2021)

SUPPLEMENTARY NOTE 1. INTERACTING ELECTRON-MAGNON MODEL

In this section, we derive the Hamiltonian of the interacting electron-magnon problem, which is valid at *low* temperatures ($T < T_{\rm C}$), when the fluctuations around the ordered phase are not too strong. In this regime, the spin operators S_i^α (with $\alpha = x, y, z$) of the localized Eu moments can be mapped, via a Holstein-Primakoff transformation, into bosonic (magnons) creation and annihilation operators that describe excitation about the ordered magnetic phase,

$$S_j^z = \hbar(S - \hat{n}_j) \ , \tag{1}$$

$$S_j^+ = S_j^x + iS_j^y = \hbar\sqrt{2S - \hat{n}_j} b_j \ , \tag{2}$$

$$S_j^- = S_{j^x} - iS_j^y = \hbar b_j^\dagger \sqrt{2S - \hat{n}_j} \ . \tag{3}$$

Here, $\hat{n}_j = b_j^\dagger b_j$ is the bosonic number operator. These expressions can be further simplified by the approximation $\sqrt{2S - \hat{n}_i} \approx \sqrt{2S}$, valid when the number of bosons excited in the system is not very large. In this case, in momentum space, we find

$$S_{\mathbf{q}}^z = \hbar S\sqrt{N}\delta_{\mathbf{q},0} - \frac{\hbar}{\sqrt{N}} \sum_{\mathbf{k}} b_{\mathbf{k}}^\dagger b_{\mathbf{q}+\mathbf{k}} \ , \tag{4}$$

$$S_{\mathbf{q}}^+ = \hbar\sqrt{2S} b_{\mathbf{q}} \ , \tag{5}$$

$$S_{\mathbf{q}}^- = \hbar\sqrt{2S} b_{-\mathbf{q}}^\dagger \ , \tag{6}$$

where

$$b_i^\dagger = \frac{1}{\sqrt{N}} \sum_{\mathbf{k}} e^{-i\mathbf{k}\cdot\mathbf{R}_i} b_{\mathbf{k}}^\dagger \ , \tag{7}$$

$$S_{\mathbf{q}}^\alpha = \frac{1}{\sqrt{N}} \sum_{i=1}^{N} e^{-i\mathbf{q}\cdot\mathbf{R}_i} S_i^\alpha \ . \tag{8}$$

Recall that $\mathbf{R}_i$ denotes the position of the N localized Eu moments in an hexagonal lattice.

Substituting Eqs.(4)-(6) into Eqs.(2)-(4) of the Methods Section we get[1]:

$$H_c = \sum_{\mathbf{k},\sigma} \xi_{\mathbf{k}} c_{\mathbf{k},\sigma}^\dagger c_{\mathbf{k},\sigma} + \sum_{\mathbf{k},\mathbf{q},\sigma} \sum_{\mathbf{r}_j} v_0 e^{-i(\mathbf{k}-\mathbf{q})\cdot\mathbf{r}_j} c_{\mathbf{k},\sigma}^\dagger c_{\mathbf{q},\sigma} \ , \tag{9}$$

$$H_f = -\frac{J_{FM}\hbar^2 S^2 N z}{2} + \sum_{\mathbf{q}} \Omega_{\mathbf{q}}^{(0)} b_{\mathbf{q}}^\dagger b_{\mathbf{q}} \ , \tag{10}$$

$$H_{cf} = \sum_{\alpha,\beta} \sum_{\mathbf{k},\mathbf{q}} \Gamma_{\alpha,\beta}(\mathbf{k},\mathbf{q}) c_{\mathbf{k}\alpha}^\dagger c_{\mathbf{q}\beta} \ . \tag{11}$$

---

[1] Because of the periodicity of the magnetic lattice $\sum\limits_{i=1}^{N} e^{-i(\mathbf{k}-\mathbf{q})\cdot\mathbf{R}_i} = N\delta_{\mathbf{k},\mathbf{q}}$.

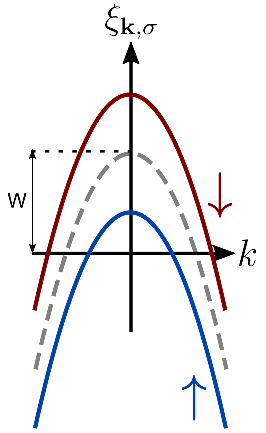

Suplementary Fig. 1: Electron dispersion of our effective model. The dashed line correspond to a spin-degenerate parabolic hole-like band (effective mass $m^* < 0$). W corresponds to the energy of the top of the band. The solid lines correspond the spin split of the band in majority (spin up) and minority (spin down) bands, which occur in the ferromagnetic phase, as discussed in the Supplementary Note 3.

Here, $\xi_{\mathbf{k}} = \hbar^2 k^2/(2m^*) + W - \mu$ is the hole-like parabolic electron dispersion, as illustrated in Supplementary Fig.1. We denote by $m^* = -m_e$ the effective electron mass, m_e is the electron rest mass, μ is the chemical potential that controls the occupation of the band, and W denotes the energy of the top of the band. Besides,

$$\Omega^{(0)}_{\mathbf{q}} = J_{FM}\hbar^2 S\left(z - 2\sum_{\boldsymbol{\delta}}\cos(\mathbf{q}\cdot\boldsymbol{\delta})\right) , \tag{12}$$

is the magnon dispersion, which will be latter approximated by a quadratic dispersion (see Supplementary Note 4), and the vector $\boldsymbol{\delta}$ connects nearest neighbor sites of the magnetic lattice. Higher-order terms in $\hat{n}_i$ gives rise to magnon-magnon interaction, which we do not take into account in this model.

The vertex of the electron-magnon interaction $\Gamma_{\alpha,\beta}(\mathbf{k},\mathbf{q})$ is the combination of four processes,

$$\Gamma_{\alpha,\beta}(\mathbf{k},\mathbf{q}) = \Gamma^{(z)}_{\alpha,\beta}(\mathbf{k},\mathbf{q}) + \Gamma^{(+)}_{\alpha,\beta}(\mathbf{k},\mathbf{q}) + \Gamma^{(-)}_{\alpha,\beta}(\mathbf{k},\mathbf{q}) + \tilde{\Gamma}_{\alpha,\beta}(\mathbf{k},\mathbf{q}) , \tag{13}$$

with

$$\Gamma^{(z)}_{\alpha,\beta}(\mathbf{k},\mathbf{q}) \equiv -\frac{J\hbar^2}{2} S\,\sigma^z_{\alpha,\beta}\,\delta_{\mathbf{k},\mathbf{q}}\ , \tag{14}$$

$$\Gamma^{(+)}_{\alpha,\beta}(\mathbf{k},\mathbf{q}) \equiv -\frac{J\hbar^2}{2}\sqrt{\frac{S}{2N}}\sigma^+_{\alpha,\beta}\,b^\dagger_{\mathbf{q}-\mathbf{k}}\ , \tag{15}$$

$$\Gamma^{(-)}_{\alpha,\beta}(\mathbf{k},\mathbf{q}) \equiv -\frac{J\hbar^2}{2}\sqrt{\frac{S}{2N}}\sigma^-_{\alpha,\beta}\,b_{\mathbf{k}-\mathbf{q}}\ , \tag{16}$$

$$\tilde{\Gamma}_{\alpha,\beta}(\mathbf{k},\mathbf{q}) \equiv \frac{J\hbar^2}{2N}\sigma^z_{\alpha,\beta}\sum_{\mathbf{k}'} b^\dagger_{\mathbf{k}'} b_{\mathbf{k}'+\mathbf{k}-\mathbf{q}}\ . \tag{17}$$

These processes are illustrated in Supplementary Fig.2. Note that $\Gamma^{(z)}$ and $\tilde{\Gamma}$ are spin-conserving processes, while $\Gamma^{(+)}$ ($\Gamma^{(-)}$) involves the creation (absorption) of a magnon and require a flip of the electron spin. In the previous equations, $\sigma^\pm = \sigma^x \pm i\sigma^y$, where σ^x, σ^y and σ^z are the Pauli matrices.

SUPPLEMENTARY NOTE 2. PERTURBATION THEORY

In this section we treat H_{cf} using standard diagrammatic techniques to investigate the effects of magnons on the electronic degrees of freedom up to the order J^2. We also address the feedback effect of the electrons in the magnon propagator up to linear order in J. The calculations performed in this section follow those of *Woolsey et al. PRB **1**, 11 (1970).*

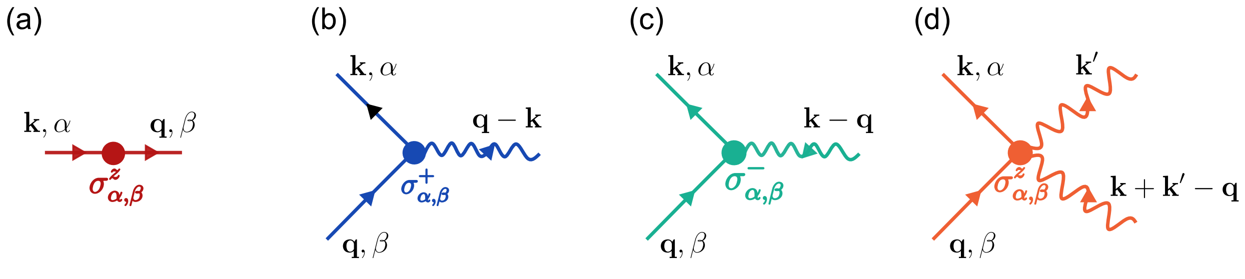

Suplementary Fig. 2: Vertex of the electron-magnon interaction. (a-d) vertexes are defined in Eqs. (14)-(17) respectively.

Bosonic degrees of freedom

The (finite-temperature T) propagator of free magnons is given by

$$\mathcal{D}^{(0)}(\mathbf{q},\nu_n) = \frac{1}{i\nu_n - \Omega^{(0)}_{\mathbf{q}}/\hbar} \ , \tag{18}$$

where $\nu_n = 2n\pi k_B T/\hbar$ (integer n) are the bosonic Matsubara frequencies and $\Omega^{(0)}(\mathbf{q})$ is the free magnon dispersion defined in Eq.(12). In lowest-order perturbation theory (RPA-like Dyson equation shown in Supplementary Fig. 3), the electron-magnon interaction promotes a energy shift in the magnon dispersion: $\Omega(\mathbf{q}) = \omega_0 + \Omega^{(0)}(\mathbf{q})$, with

$$\omega_0 = J\hbar^2 \frac{\mathcal{V}}{2}(n_\uparrow - n_\downarrow) \ . \tag{19}$$

Here, $n_\uparrow$ ($n_\downarrow$) denotes the density of electrons with spin projection up (down). Besides, $\mathcal{V} = V/N$ is the volume of the direct magnetic cell and V is the total volume of the system.

Electronic degrees of freedom

To investigate the effects of magnons on the electrons, we start by calculating the electronic Green's function dressed by self-energy insertions that are built using only the vertex $\Gamma^{(z)}$, as shown in Supplementary Fig.4(a),

$$\mathcal{G}^{(A,1)}_{\alpha,\beta}(\mathbf{k},\omega_n) = \left[\mathcal{G}^{(0)\,-1}_{\alpha,\beta}(\mathbf{k},\omega_n) - \Sigma^{(A,1)}_{\alpha,\beta}(\mathbf{k},\omega_n)\right]^{-1} \ , \tag{20}$$

where

$$\mathcal{G}^{(0)}_{\alpha,\beta}(\mathbf{k},\omega_n) = \frac{\delta_{\alpha,\beta}}{i\omega_n - \xi_{\mathbf{k}}/\hbar} \tag{21}$$

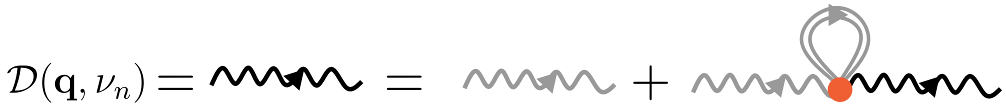

Suplementary Fig. 3: Dyson equation for the magnon propagator. The interaction vertex follow the same color code as in Supplementary Fig.2. The gray wiggle line correspond to the bare magnon propagator $\mathcal{D}^{(0)}(\mathbf{q},\nu_n)$ defined in Eq.(18), while the black wiggle line represent the dressed bosonic propagator up to first order in J. The solid gray double line correspond to the first-order fermion Green's function (see next section).

is the bare electron Green's function, with $\omega_n = (2n+1)\pi k_B T/\hbar$ (n integer) denoting the fermionic Matsubara frequency, and

$$\Sigma^{(A,1)}_{\alpha,\beta}(\mathbf{k},\omega_n) = -\frac{\gamma_0}{\hbar}\sigma^{(z)}_{\alpha,\beta} \; , \tag{22}$$

where we define $\gamma_0 \equiv J\hbar^2 S/2$. The Dyson's equation (20) gives us the electron Green's function in first-order perturbation theory,

$$\mathcal{G}^{(A,1)}_{\uparrow,\uparrow}(\mathbf{k},\omega_n) = \frac{1}{i\omega_n - (\xi_{\mathbf{k}} - \gamma_0)/\hbar} \; , \tag{23}$$

$$\mathcal{G}^{(A,1)}_{\downarrow,\downarrow}(\mathbf{k},\omega_n) = \frac{1}{i\omega_n - (\xi_{\mathbf{k}} + \gamma_0)/\hbar} \; , \tag{24}$$

$$\mathcal{G}^{(A,1)}_{\uparrow,\downarrow}(\mathbf{k},\omega_n) = \mathcal{G}^{(0)}_{\downarrow,\uparrow}(\mathbf{k},\omega_n) = 0 \; , \tag{25}$$

which tell us that the effect of the electron-magnon interaction is to generate a Zeeman-like energy shift of the electron bands $\xi_{\mathbf{k}} \to \xi_{\mathbf{k},\sigma} = \xi_{\mathbf{k}} - \gamma_0\sigma$, where we identify $\uparrow = +$ and $\downarrow = -$. As discussed in the main text, this causes the spin-up band to shift down in energy and it becomes a *majority band*, since it can now accommodate more electrons. The spin-down band, on the other hand, shifts up in energy and becomes and corresponds to a *minority band*.

This result can be further improved. The shift of the electron bands cannot be really a constant, but it is rather a temperature-dependent function. The reasoning is the following: as the temperature increases, more spin-flip processes take place, causing ground-state

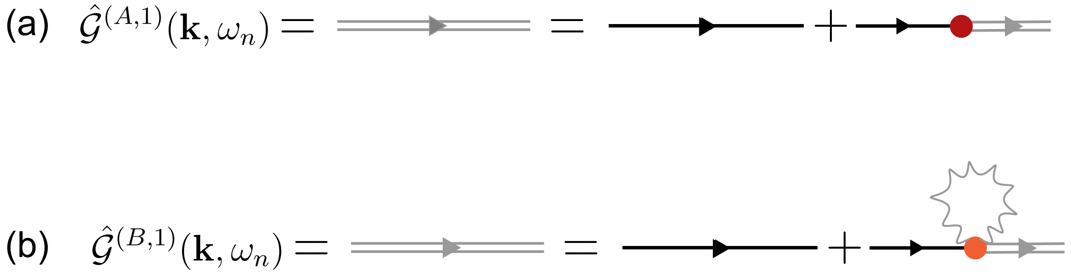

Suplementary Fig. 4: Dyson equations. (a) for the electron Green's function up to first order in J corresponding to equation (20) (b) same as (a) but for equation (26) . $\hat{\mathcal{G}}$ denotes a matrix in spin-space whose matrix elements are $\mathcal{G}_{\alpha,\beta}$ specified in the text. The interaction vertex follow the same color code as in Supplementary Fig.2. The gray wiggle line correspond to the bare magnon propagator defined in Eq.(18), while the black solid line represent bare fermion Green's function defined in Eq.(21).

magnetization to decrease. Equivalently, more magnons are introduced in the system as temperature increases, until the ferromagnetic order melts at the transition temperature $T = T_{\rm C}$. As a consequence, the effective magnetic field felt by the electrons decreases with temperature, and so does the band-splitting. This effect is captured by another set of first-order diagrams involving the vertex $\tilde{\Gamma}_{\alpha,\beta}(\mathbf{k},\mathbf{q})$ defined in Eq.(17), as illustrated in Supplementary Fig.4 (b). The Dyson equation we have to solve, in this case, is

$$\mathcal{G}^{(B,1)}_{\alpha,\beta}(\mathbf{k},\omega_n) = \left[\mathcal{G}^{(0)\,-1}_{\alpha,\beta}(\mathbf{k},\omega_n) - \Sigma^{(B,1)}_{\alpha,\beta}(\mathbf{k},\omega_n)\right]^{-1} , \tag{26}$$

$$\Sigma^{(B,1)}_{\alpha,\beta}(\mathbf{k},\omega_n) = -\frac{k_B T}{\hbar^2}\frac{J\hbar^2}{2N} V \sigma^{(z)}_{\alpha,\beta} \sum_{n'} \int \frac{d^3q}{(2\pi)^3} \mathcal{D}^{(0)}(\mathbf{q},\nu'_n) , \tag{27}$$

where $\mathcal{D}^{(0)}(\mathbf{q},\nu_n)$ is the bosonic propagator defined in Eq.(18).

The Matubara sum in Eq.(30) can be easily calculated:

$$\frac{k_B T}{\hbar} \sum_n \frac{1}{i\nu_n - \Omega^{(0)}_{\mathbf{q}}/\hbar} = \int_{\mathcal{C}} \frac{dz}{2\pi i} \frac{1}{e^{z/(k_B T)} - 1} \frac{1}{z - \Omega^{(0)}_{\mathbf{q}}} = -n_B(\Omega^{(0)}_{\mathbf{q}}) , \tag{28}$$

where $n_B(\epsilon) = \left(e^{\epsilon/(k_B T)} - 1\right)^{-1}$ is the Bose-Einstein distribution function. The remaining momentum integration can also be evaluated analytically when the boson dispersion is approximated to a parabola [1],

$$\frac{V}{N} \int \frac{d^3q}{(2\pi)^3} n_B(\Omega^{(0)}_{\mathbf{q}}) = \frac{1}{8} \left(\frac{k_B T}{J_{FM}\hbar^2 S\pi}\right)^{3/2} \zeta(3/2) . \tag{29}$$

Therefore,

$$\Sigma^{(B,1)}_{\alpha,\beta}(\mathbf{k},\omega_n) = \frac{1}{\hbar}\frac{J\hbar^2}{2}\sigma^{(z)}_{\alpha,\beta}\frac{1}{8}\left(\frac{k_B T}{J_{FM}\hbar^2 S\pi}\right)^{3/2} \zeta(3/2) , \tag{30}$$

where $\zeta(x)$ is the Riemann zeta function.

Combining Eqs.(22) and (30), we obtain

$$\Sigma^{(1)}_{\alpha,\beta}(\mathbf{k},\omega_n) = -\frac{1}{\hbar}\frac{J\hbar^2}{2}\langle S_z\rangle , \tag{31}$$

$$\langle S_z\rangle = S - \frac{1}{8}\left(\frac{k_B T}{J_{FM} S\hbar^2\pi}\right)^{3/2} \zeta(3/2) . \tag{32}$$

The corresponding dressed first-order Green's function has the same form of Eqs.(23)-(25), but with a T-dependent shift of the bands $\xi_{\mathbf{k},\sigma} = \xi_{\mathbf{k}} - \gamma(T)\sigma$, with

$$\gamma(T) \equiv \frac{J\hbar^2}{2}\left(S - \frac{1}{8}\left(\frac{k_B T}{J_{FM}\hbar^2 S\pi}\right)^{3/2} \zeta(3/2)\right) . \tag{33}$$

Importantly, when $T = T_\mathrm{C}$, $\gamma(T_\mathrm{C}) = 0$, which allow us to identify

$$J_{FM}\hbar^2 S = \frac{k_B T_\mathrm{C}}{\pi}\left(\frac{\zeta(3/2)}{8S}\right)^{2/3} . \tag{34}$$

For $EuCd_2As_2$, $T_\mathrm{C} \approx 26K$ and $S = 7/2$, therefore $J_{FM}S\hbar^2 \approx 0.15meV$.

We now focus on second-order perturbation theory, which, as we will shortly show, leads to a spin-dependent scattering of the electrons due to magnons, which we call *magnetic scattering rate*. Diagrams involving the $\Gamma^{(+)}_{\alpha,\beta}(\mathbf{k},\mathbf{q})$ and $\Gamma^{(-)}_{\alpha,\beta}(\mathbf{k},\mathbf{q})$ [see Eqs.(15) and (16)] requires at least two vertex, so the magnon absorption and emission lines can be combined. Below, we consider only the rainbow diagrams shown in Supplementary Fig.5. The second-order electronic Green's function is then given by the Dyson equation

$$\mathcal{G}^{(2)}_{\alpha,\beta}(\mathbf{k},\omega_n) = \left[\mathcal{G}^{(0)\,-1}_{\alpha,\beta}(\mathbf{k},\omega_n) - \Sigma^{(2)}_{\alpha,\beta}(\mathbf{k},\omega_n)\right]^{-1} , \tag{35}$$

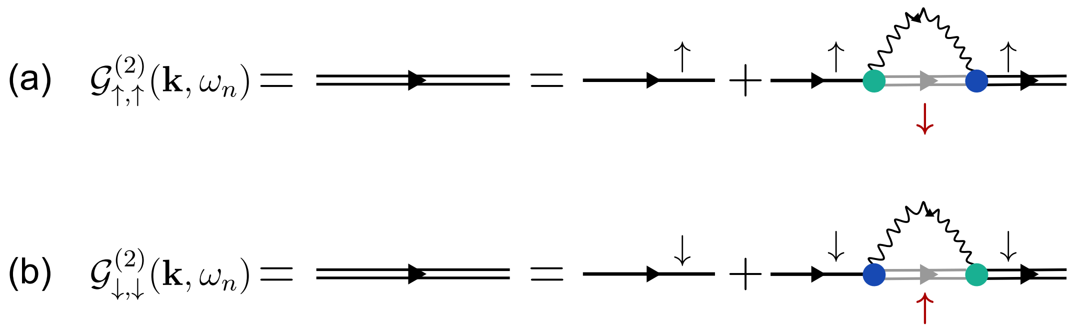

Suplementary Fig. 5: Dyson equations (35) for the electron Green's function up to second order in J. (a) Green's function for majority carriers. (b) Green's function for minority carriers. The interaction vertex follow the same color code as in Supplementary Fig.2. The black wiggle line correspond to the dressed magnon propagator and the single solid line correspond to the bare electron Green's function defined in Eq.(21). The double gray (black) solid line, on the other hand, correspond to the electron Green's function up to first (second) order in J.

with

$$\Sigma^{(2)}_{\uparrow,\uparrow}(\mathbf{k},\omega_n) = -\frac{k_B T}{\hbar^3} V \sum_{n'} \sum_{\alpha',\beta'} \int \frac{d^3q}{(2\pi)^3} \mathcal{D}(\mathbf{q}-\mathbf{k},\omega'_n-\omega_n) \Gamma^{(+)}_{\alpha,\alpha'}(\mathbf{k},\mathbf{q}) \mathcal{G}^{(1)}_{\alpha'\beta'}(\mathbf{q},\omega'_n) \Gamma^{(-)}_{\beta',\beta}(\mathbf{q},\mathbf{k}) \ , \tag{36}$$

$$\Sigma^{(2)}_{\downarrow,\downarrow}(\mathbf{k},\omega_n) = -\frac{k_B T}{\hbar^3} V \sum_{n'} \sum_{\alpha',\beta'} \int \frac{d^3q}{(2\pi)^3} \mathcal{D}(\mathbf{k}-\mathbf{q},\omega_n-\omega'_n) \Gamma^{(-)}_{\alpha,\alpha'}(\mathbf{k},\mathbf{q}) \mathcal{G}^{(1)}_{\alpha'\beta'}(\mathbf{q},\omega'_n) \Gamma^{(+)}_{\beta',\beta}(\mathbf{q},\mathbf{k}) \ . \tag{37}$$

Here, $\mathcal{D}(\mathbf{q}-\mathbf{k},\omega'_n-\omega_n)$ is the magnon propagator with the same form of Eq.(18), but with the remormalized dispersion $\Omega_\mathbf{q} = \omega_0 + \Omega^{(0)}_\mathbf{q}$.

Substituting Eqs.(15), (16), (18) and (21) into Eqs.(36)-(37), we obtain, after evaluating the Matsubara sum,

$$\Sigma^{(2)}_{\uparrow,\uparrow}(\mathbf{k},\omega_n) = V \frac{J^2\hbar^4 S}{2N} \frac{1}{\hbar^2} \int \frac{d^3q}{(2\pi)^3} \frac{n_F(\xi_{\mathbf{q},\downarrow}) + n_B(\Omega_{\mathbf{q}-\mathbf{k}})}{i\omega_n - (\xi_{\mathbf{q},\downarrow}/\hbar) + (\Omega_{\mathbf{q}-\mathbf{k}}/\hbar)} \tag{38}$$

$$\Sigma^{(2)}_{\downarrow,\downarrow}(\mathbf{k},\omega_n) = V \frac{J^2\hbar^4 S}{2N} \frac{1}{\hbar^2} \int \frac{d^3q}{(2\pi)^3} \frac{1 + n_B(\Omega_{\mathbf{k}-\mathbf{q}}) - n_F(\xi_{\mathbf{q},\uparrow})}{i\omega_n - (\xi_{\mathbf{q},\uparrow}/\hbar) - (\Omega_{\mathbf{k}-\mathbf{q}}/\hbar)} \ . \tag{39}$$

From these finite-T self-energies, we can obtain the magnetic scattering rates after performing the analytic continuation $i\omega_n \to \omega + i\text{sgn}(\omega_n)\eta$, with $\eta \to 0^+$, and using the definition of the quasi-particle scattering rate [2]

$$\frac{1}{2\tau_\mathbf{k}} = -\text{sgn}(\omega_n)\ \text{Im}\Sigma(\mathbf{k},\omega + i\text{sgn}(\omega_n)\eta)|_{\omega=\xi_k/\hbar} \ . \tag{40}$$

The fact that we have different self-energies for spin-up and spin-down electrons reflect in spin-dependent magnetic scattering rates:

$$\Gamma_{mag,\mathbf{k},\uparrow} \equiv \frac{1}{2\tau_{mag,\mathbf{k},\uparrow}} = \frac{\pi J^2\hbar^4 S\mathcal{V}}{2\hbar} \int \frac{d^3q}{(2\pi)^3} \left[n_F(\xi_{\mathbf{q},\downarrow}) + n_B(\Omega_{\mathbf{q}-\mathbf{k}})\right] \delta\left(\xi_{\mathbf{k},\uparrow} + \Omega_{\mathbf{q}-\mathbf{k}} - \xi_{\mathbf{q},\downarrow}\right) \tag{41}$$

$$\Gamma_{mag,\mathbf{k},\downarrow} \equiv \frac{1}{2\tau_{mag,\mathbf{k},\downarrow}} = \frac{\pi J^2\hbar^4 S\mathcal{V}}{2\hbar} \int \frac{d^3q}{(2\pi)^3} \left[1 + n_B(\Omega_{\mathbf{k}-\mathbf{q}}) - n_F(\xi_{\mathbf{q},\uparrow})\right] \delta\left(\xi_{\mathbf{k},\downarrow} - \Omega_{\mathbf{k}-\mathbf{q}} - \xi_{\mathbf{q},\uparrow}\right) \ , \tag{42}$$

Making the change of variables $\mathbf{q}-\mathbf{k} \to \mathbf{q}$ and $\mathbf{k}-\mathbf{q} \to \mathbf{q}$ in Eq.(41) and Eq.(42), respectively, we find

$$\Gamma_{mag,\mathbf{k},\uparrow} = \frac{\pi J^2\hbar^4 S\mathcal{V}}{2\hbar} \int \frac{d^3q}{(2\pi)^3} \left[n_F(\xi_{\mathbf{q}+\mathbf{k},\downarrow}) + n_B(\Omega_\mathbf{q})\right] \delta\left(\xi_{\mathbf{k},\uparrow} + \Omega_\mathbf{q} - \xi_{\mathbf{q}+\mathbf{k},\downarrow}\right) \tag{43}$$

$$\Gamma_{mag,\mathbf{k},\downarrow} = \frac{\pi J^2\hbar^4 S\mathcal{V}}{2\hbar} \int \frac{d^3q}{(2\pi)^3} \left[1 + n_B(\Omega_\mathbf{q}) - n_F(\xi_{\mathbf{k}-\mathbf{q},\uparrow})\right] \delta\left(\xi_{\mathbf{k},\downarrow} - \Omega_\mathbf{q} - \xi_{\mathbf{k}-\mathbf{q},\uparrow}\right) \ , \tag{44}$$

For the results shown in the main text, we evaluate the integrals in Eqs.(43)-(44) at the Fermi level assuming a quadratic dispersion for the bosons,

$$\Omega_{\mathbf{q}} = \omega_0 + \frac{\hbar^2 q^2}{2M} \ , \tag{45}$$

where M denotes the effective magnon mass [see Supplementary Note 4]. In this case, there is a natural upper cutoff for the momentum integral, $0 \leq q \leq q_{max}$, where $4\pi q_{max}^3/3 = V_{1BZ}$ and V_{1BZ} denotes the volume of the first Brillouin zone of the magnetic lattice, above which the parabolic approximation breaks down.

Note that the magnetic scattering rate for spin-up electrons depend on the phase space available for scattering to intermediary spin-down states and vice versa. Since, for hole-like bands, the Fermi surface of spin-down electrons (minority) is larger than that of spin-up spins (majority), it follows that $\Gamma_{mag,\uparrow} > \Gamma_{mag,\downarrow}$. The result would be the opposite for electron-like bands ($m^* > 0$).

SUPPLEMENTARY NOTE 3. IMPURITY SCATTERING

Another channel for electron scattering in $EuCd_2As_2$ is the impurity scattering. To calculate the *impurity scattering rate*, we consider a total of N_{imp} non-magnetic impurities placed at random positions $\boldsymbol{\tau}_j$. For simplicity, we focus on the case of point-like disorder, so the impurity potential felt by the electrons takes the form of the second term in Eq.(9). Disorder then dresses the electron Green's function according to

$$\mathcal{G}_{\alpha,\beta}(\mathbf{k},\omega_n) = \left[\mathcal{G}^{(1)\,-1}_{\alpha,\beta}(\mathbf{k},\omega_n) - \Sigma^{(imp)}_{\alpha,\beta}(\mathbf{k},\omega_n)\right]^{-1} \ , \tag{46}$$

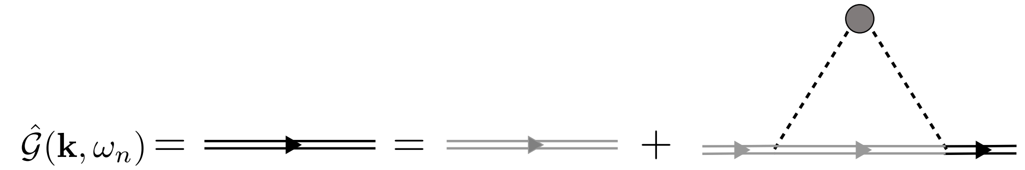

Suplementary Fig. 6: Dyson equations (46) for the electron Green's function dressed by disorder according to the first-order Born approximation. The gray double solid line correspond to the fist-order Green's function $\hat{\mathcal{G}}^{A,1}(\mathbf{k},\omega_n) + \hat{\mathcal{G}}^{B,1}(\mathbf{k},\omega_n)$. The dotted line represent the impurity potential v_0 and the solid gray circle signifies the impurity density n_{imp}.

where the impurity self-energy is obtained via standard Born-approximation, as illustrated in Supplementary Fig.3. Here, $\mathcal{G}^{(1)}_{\alpha,\beta}(\mathbf{k},\omega_n)$ is the first-order Green's function calculated in Supplementary Note 2. Because of the spin-split of the band in the ferromagnetic phase, impurities scatter spin-up electrons and spin-down electrons differently. As we will shortly see, as in the case of the magnetic scattering rate, this is related to different phase spaces of spin-up and spin-down electron.

The impurity self-energy is given by

$$\Sigma^{(imp)}_{\alpha,\beta}(\mathbf{k},\omega_n) = \delta_{\alpha,\beta}\frac{n_{imp}|v_0|^2}{\hbar^2}\frac{1}{V}\sum_{\mathbf{k}'}\frac{1}{i\omega_n - (\xi_{\mathbf{k}',\alpha}/\hbar)} \ . \tag{47}$$

Performing the analytic continuation as in Supplementary Note 2, we find

$$\Gamma_{imp,\mathbf{k},\sigma} \equiv \frac{1}{2\tau_{imp,\mathbf{k},\sigma}} = \frac{\pi n_{imp}|v_0|^2}{\hbar}\int \frac{d^3k'}{(2\pi)^3}\delta(\xi_{\mathbf{k},\sigma} - \xi_{\mathbf{k}',\sigma}) \ . \tag{48}$$

For parabolic bands, Eq.(48) can be calculated analytically, yielding, at the Fermi level,

$$\Gamma_{imp,\sigma} = n_{imp}|v_0|^2\frac{2m}{4\pi\hbar^3}k_{F,\sigma} \ , \tag{49}$$

where $k_{F,\sigma}$ is the Fermi-level momentum of the spin-σ band. From Eq.(49), it becomes evident that the impurity scattering rate is larger for the band that has the larger Fermi surface. In the case of hole-like bands, this means, in contrast with the magnetic scattering rate, that $\Gamma_{imp,\uparrow} < \Gamma_{imp,\downarrow}$.

SUPPLEMENTARY NOTE 4. EFFECTIVE MAGNON MASS

We mentioned before that we use a parabolic approximation for the energy dispersion of the magnons in order to calculate the magnetic scattering rate [see Eq.(45)]. Here, we show how we estimated the effective magnon mass M for the case of $EuCd_2As_2$.

Expanding Eq.(12) up to second order in $\mathbf{q}\cdot\boldsymbol{\delta}$, we find

$$\Omega^{(0)}_{\mathbf{q}} = J_{FM}\hbar^2 S\left[(\mathbf{q}\cdot\boldsymbol{\delta}_1)^2 + (\mathbf{q}\cdot\boldsymbol{\delta}_2)^2 + (\mathbf{q}\cdot\boldsymbol{\delta}_3)^2\right] \ , \tag{50}$$

where $\boldsymbol{\delta}_i$ $(i = 1, 2, 3)$ are the vectors connecting the first-neighbor sites of the magnetic lattice. The Eu atoms in $EuCd_2As_2$ form an simple hexagonal lattice, for which

$$\boldsymbol{\delta}_1 = a\hat{x} \ , \tag{51}$$

$$\boldsymbol{\delta}_2 = \frac{a}{2}\hat{x} + \frac{\sqrt{3}a}{2}\hat{y} \ , \tag{52}$$

$$\boldsymbol{\delta}_3 = c\hat{z} \ , \tag{53}$$

with $a \approx 4.43$ Åand $c \approx 7.32$ Å. Substituting Eqs.(51)-(53) into Eq.(50), we obtain, neglecting the cross terms

$$\Omega^{(0)}_{\mathbf{q}} \approx J_{FM}\hbar^2 S\left(\frac{5a^2}{4}q_x^2 + \frac{3a^2}{4}q_y^2 + c^2 q_z^2\right) . \tag{54}$$

For simplicity, we further approximate he boson dispersion to be isotropic. In this case,

$$\Omega^{(0)}_{\mathbf{q}} \equiv J_{FM}\hbar^2 S \left\langle a^2\right\rangle q^2 = \frac{\hbar^2 q^2}{2M} , \tag{55}$$

where $\langle a^2\rangle = (2a^2 + c^2)/3 \approx 3.1 \times 10^{-19}$ m^{-1}, and therefore $M = 1/(2J_{FM}S)\langle a^2\rangle \approx 7.5 \times 10^{-28}$ Kg.

SUPPLEMENTARY NOTE 5. CARRIER DENSITY

Hall conductivity data gives a carrier density of $n_H = 2.35 \times 10^{26}$ m^{-3} for $EuCd_2As_2$, where the majority of carriers are holes. We use a carrier density $n = 2n_H/3$ to calculate the chemical potential $\tilde{\mu} = W - \mu$ self-consistently trough $N_h = N_{tot} - N_e$, where N_h is the number of the holes in the system, which is obtained by subtracting from the total number of states that fits in a parabolic band with band-width Λ,

$$N_{tot} = N_{\uparrow}^{(tot)} + N_{\downarrow}^{(tot)} = \frac{V}{4\pi^2}\frac{(2m)^{3/2}}{\hbar^3}\left[\int_{-\Lambda}^{\tilde{\mu}-\gamma} d\tilde{\xi}\sqrt{\tilde{\mu}-\gamma-\tilde{\xi}} + \int_{-\Lambda}^{\tilde{\mu}+\gamma} d\tilde{\xi}\sqrt{\tilde{\mu}+\gamma-\tilde{\xi}}\right] , \tag{56}$$

from the total number of electrons

$$N_e = N_{e,\uparrow}+N_{e,\downarrow} = \frac{V}{4\pi^2}\frac{(2m)^{3/2}}{\hbar^3}\left[\int_{-\Lambda}^{\tilde{\mu}-\gamma} d\tilde{\xi}\sqrt{\tilde{\mu}-\gamma-\tilde{\xi}}\frac{1}{e^{\tilde{\xi}/k_BT}+1} + \int_{-\Lambda}^{\tilde{\mu}+\gamma} d\tilde{\xi}\sqrt{\tilde{\mu}+\gamma-\tilde{\xi}}\frac{1}{e^{\tilde{\xi}/k_BT}+1}\right] . \tag{57}$$

In Eqs.(56) and (57), $\gamma = \gamma(T)$ defined in Eq.(33). The resulting density of holes $n_h = N_h/V$ is independent of the band width, as long as $\Lambda \gg \tilde{\mu} \pm \gamma$.

SUPPLEMENTARY NOTE 6. COMPLEMENTARY FIGURE

We showed that the impurity scattering dominates over magnetic scattering at low temperatures in $EuCd_2As_2$. As a consequence, $\Gamma_{\downarrow} > \Gamma_{\uparrow}$ (recalling that $\Gamma_\sigma = \Gamma_{mag,\sigma} + \Gamma_{imp,\sigma}$) for small T, as observed in ARPES data. In the figures shown in the main text of this manuscript, we set $JS\hbar^2 = 150meV$, which we estimated using the experimentally observed

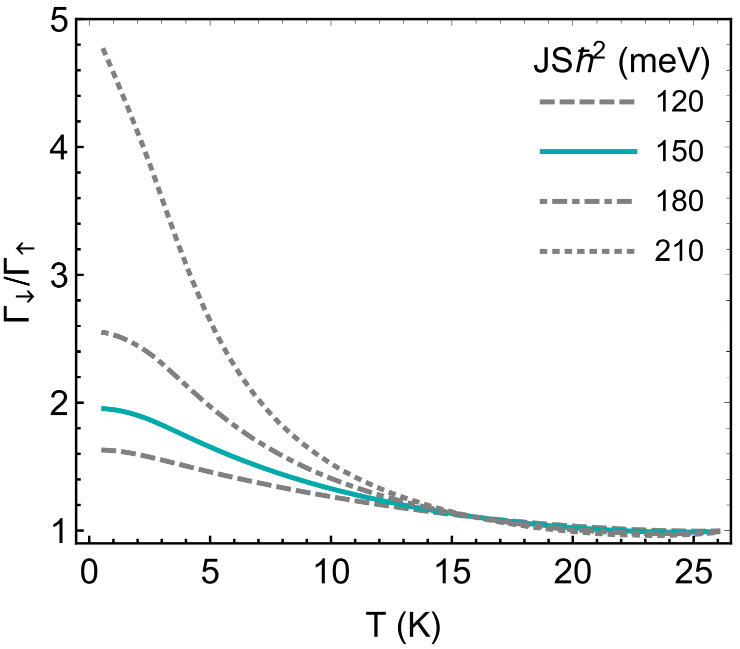

Suplementary Fig. 7: Ratio of the total scattering rates $\Gamma_\downarrow/\Gamma_\uparrow$ as function of temperature for different values of J. We set $n = n_0 = 2.35 \times 10^{26} m^{-3}$ and $J_{FM} S\hbar^2 = 0.15 meV$. The values of J were carefully chosen so that both majority and minority bands crosses the Fermi level.

splitting of minority and majority bands. However, there are other effects, such as lattice deformation, that could contribute to the band shift. This gives a natural uncertainty for the estimated value of J, and to complement our results, we show here the ratio $\Gamma_\downarrow/\Gamma_\uparrow$ as a function of temperature for different values of J. We see that the sharpening of the inner band is more pronounced for larger values of the coupling between the itinerant electron and the localized moments.

SUPPLEMENTARY NOTE 7. IMPACT OF THE PARAMETERS OF THE MODEL ON Γ_σ.

We systematically studied the effect of the Kondo coupling J, carrier density n, impurity strength and density $n_{imp} |v_0|^2$, and effective electron mass $m*$ on the total scattering rate $\Gamma_\sigma = \Gamma_{imp,\sigma} + \Gamma_{mag,\sigma}$. Varying one of these parameters at a time, while keeping the others fixed, we found that:

- Larger values of J make Γ_σ more steep with increasing T and enhances the ratio $\Gamma_\downarrow/\Gamma_\uparrow$

at low temperatures.

- Larger values of $n_{imp}\left|v_0\right|^2$ make the curves Γ_σ flatter as a function of T. It does not show a significant impact on the $\Gamma_\downarrow/\Gamma_\uparrow$ ratio.

- Larger values of the carrier (hole) density make the curves Γ_σ more flat and reduces the $\Gamma_\downarrow/\Gamma_\uparrow$ ratio at low temperature.

- Increasing the magnitude of the effective electronic mass has a similar effect to increasing J.

Note that we did not vary the Heisenberg coupling J_{FM} neither the magnon mass M, since there is less uncertainty related to the experimental quantities fixing these parameters. For instance, J_{FM} is directly related to the ferromagnetic transition temperature T_c, and the magnon mass is set by the lattice parameter and J_{FM}.

Based on the analysis above, we conclude that stronger Kondo coupling, lower hole densities and larger impurity strength and/or density are ideal to recover the flat character of the minority band linewidth with decreasing temperature and the experimental ratio $\Gamma_\downarrow/\Gamma_\uparrow$ at low T. The theoretical scattering rate Γ_σ for a set of parameters different than those used in the main text is shown in Supplementary Fig. 8. Note that in contrast to Supplementary Fig.4(c) in the main text, $\Gamma_\downarrow/\Gamma_\uparrow$ becomes smaller than one for T in the vicinity of T_{C}. That is because here $n_{imp}\left|v_0\right|^2$ is smaller and therefore the impurity scattering starts starts dominating Γ_σ at lower temperatures, resulting in $\Gamma_\uparrow > \Gamma_\downarrow$ in the vicinity of the ferromagnetic transition.

SUPPLEMENTARY REFERENCES

* These two authors contributed equally

† kaminski@ameslab.gov

[1] A. Auerbach, *Interacting Electrons and Quantum Magnetism* (Springer, New York, 1994).

[2] H. Bruus and K. Flensberg, *Many-Body Quantum Theory in Condensed Matter Physics: An Introduction* (OUP Oxford, 2004).

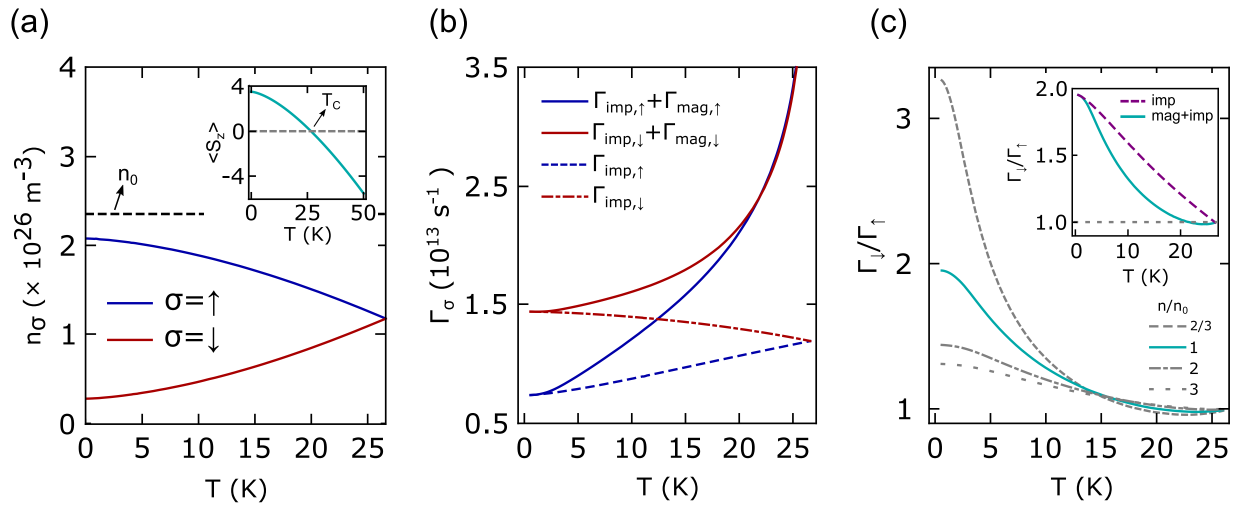

Suplementary Fig. 8: **Theoretical model results.** (a) Spin resolved carrier (holes) density n_σ as a function of temperature T below the magnetic transition $T_\mathrm{C} \approx 26$ K in the theoretical model with $n_H = n_\uparrow + n_\downarrow = 2.35 \times 10^{26}$ m^{-3} extracted from Hall measurements, $JS\hbar^2 = 150$ meV (instead of $J = 165$ meV), and $J_\mathrm{FM}S\hbar^2 = 0.15$ meV. Inset shows average z-component $\langle S_z\rangle(T)$ of Eu spins versus T, used to estimate FM coupling constant $J_\mathrm{FM}S$ from $\langle S_z\rangle(T_\mathrm{C}) = 0$. (b) Total minority and majority scattering rates $\Gamma_\downarrow$ and $\Gamma_\uparrow$ (solid) as a function of T, where $\Gamma_\sigma = \Gamma_{\mathrm{imp},\sigma} + \Gamma_{\mathrm{mag},\sigma}$. Dashed lines show impurity scattering contributions, which dominate at low T. Upturn close to T_C is caused by scattering with magnons that proliferate at the phase transition. (c) Ratio of minority over majority quasiparticle scattering rates, $\Gamma_\downarrow/\Gamma_\uparrow$, as a function of T for different carrier densities n/n_H. The ratio increases for decreasing T and n, and is larger than unity except close to T_C. Inset compares ratio of total scattering rates (solid) to ratio obtained from impurity scattering only (dashed) for $n = n_H$.
